# Case Report: Life-threatening hypercalcemia associated with MMR-deficient endometrial carcinoma secreting parathyroid hormone

**DOI:** 10.3389/fendo.2023.1125822

**Published:** 2023-01-31

**Authors:** Huazhen Liu, Dan Gao, Yongfa Huang, Ji Li, Mengyun Zhao, Zhaohui Lu, Ya Hu, Tao Wang, Yingqiang Zhang, Wenze Wang, Dongyan Cao

**Affiliations:** ^1^ Department of Ultrasound, Peking Union Medical College Hospital, Chinese Academy of Medical Sciences and Peking Union Medical College, Beijing, China; ^2^ Department of Pathology, Peking Union Medical College Hospital, Chinese Academy of Medical Sciences and Peking Union Medical College, Beijing, China; ^3^ Department of Pathology, Hongqi Hospital Affiliated to Mudanjiang Medical University, Mudanjiang, China; ^4^ Liver Transplantation Center, Beijing Friendship Hospital, Capital Medical University, National Clinical Research Center for Digestive Diseases, Beijing, China; ^5^ Clinical Center for Pediatric Liver Transplantation, Capital Medical University, Beijing, China; ^6^ Department of Pathology, Molecular Pathology Research Center, Peking Union Medical College Hospital, Chinese Academy of Medical Sciences and Peking Union Medical College, Beijing, China; ^7^ Department of Anesthesiology, Peking Union Medical College Hospital, Chinese Academy of Medical Sciences and Peking Union Medical College, Beijing, China; ^8^ Department of General Surgery, Peking Union Medical College Hospital, Chinese Academy of Medical Sciences and Peking Union Medical College, Beijing, China; ^9^ Department of Obstetrics and Gynecology, Peking Union Medical College Hospital, Chinese Academy of Medical Sciences & Peking Union Medical College, Beijing, China; ^10^ Department of Nuclear Medicine, Peking Union Medical College Hospital, Chinese Academy of Medical Sciences & Peking Union Medical College, Beijing, China; ^11^ Beijing Key Laboratory of Molecular Targeted Diagnosis and Therapy in Nuclear Medicine, Beijing, China

**Keywords:** hypercalcemia, ectopic, parathyroid hormone, endometrial carcinoma, MMR-deficient

## Abstract

Ectopic secretion of parathyroid hormone (PTH) is a rare cause of hypercalcemia in malignancy patients. A 56-year-old woman with life-threatening hypercalcemia was caused by poorly-differentiated endometrial carcinoma secreting PTH with concomitant nodular goiter mimic parathyroid tumors. The elevated level of PTH and calcium decreased immediately after cytoreductive surgery (CRS). The pathology confirmed mismatch repair (MMR)-deficient endometrial carcinoma with PTH expression. The patient received four-course chemotherapy and one-course immunotherapy after CRS. The disease progression led to multiple organ failure and death about five months after CRS. To our knowledge, this is the first case of hypercalcemia caused by MMR-deficient endometrial carcinoma with ectopic PTH secreting and the first report of malignancy associated hypercalcemia complicated with nodular goiter.

## 1 Introduction

Hypercalcemia occurs in 20-30% of cancer patients during the disease course (1). The most common cause of malignancy-associated hypercalcemia (MAH) was humoral hypercalcemia of malignancy caused by PTHrP (parathyroid hormone (PTH)-related protein) with a frequency of 80%, while the rarest cause being an ectopic secretion of PTH by malignancy (<1%) (1). Quantification of serum PTH is crucial in the differentiation of MAH (2). It is reported that gynecologic neoplasms were responsible for approximately 20% of MAH, predominantly due to humoral mechanisms (3).

Here, we presented a case of high-grade mismatch repair (MMR)-deficient endometrial carcinoma with hypercalcemia and elevated PTH due to ectopic PTH secretion. To our knowledge, only one case of ectopic PTH and hypercalcemia caused by endometrial carcinoma with an absence of MMR test has been reported previously (4).

## 2 Case presentation

A 56-year-old woman with hypercalcemia and pancreatitis was referred to our hospital for diagnosis and treatment. The patient reported persistent upper abdominal pain, accompanied by nausea, vomiting, and diarrhea one month ago. She suffered from hypersomnia one week ago. Past medical history and family history were insignificant. Physical findings included the tenderness of the epigastric region.

**Figure 1:**
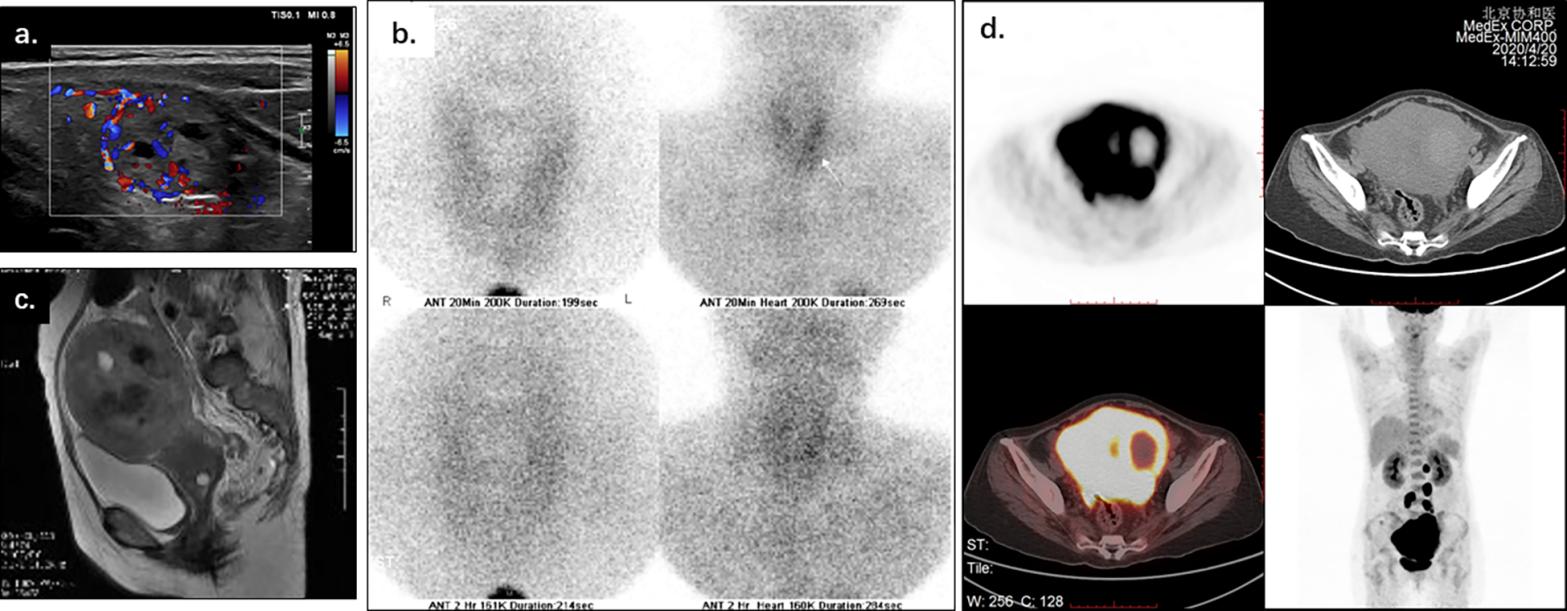
Thyroid ultrasonography, 99m Tc-MIBG, pelvic MRI, and PET-CT of a 56-year-old woman with life-threatening hypercalcemia. Thyroid ultrasonography **(A)** and 99m Tc-MIBG **(B)** showed a nodule inferior the left thyroid lobe, which may origin from parathyroid. Pelvic MRI **(C)** showed the uterus enlarged, with disrupted junctional zone, thickened myometrium, and abnormal signals in the uterine cavity. PET-CT **(D)** showed the irregularly enlarged uterus with increased SUV value (max to 15.7), as while multiple enlarged lymph nodes along the uterus, abdominal aorta, and bilateral common iliac arteries (SUVmax 15.5).

Upon examination, a distinct elevation of serum calcium (13.84mg/dl, reference range: 8.52-10.80mg/dl), and PTH (1013.1pg/ml, reference range: 12-69pg/ml) was observed. Thyroid ultrasonography and 99m Tc-MIBG (**Figures 1A, B**) showed a nodule in the lower pole of the left thyroid lobe, which may origin from parathyroid. Besides, the diagnosis of pancreatitis was confirmed base on the elevation of serum amylase (1222.5U/L dropped to 156U/L, reference range: 25-115U/L) and abdominal CT imaging (showing the diffuse enlargement of pancreas with peripancreatic inflammation). Acute renal insufficiency was diagnosed with creatine increased rapidly from normal range to 233μmol/L.

The patient received treatment for pancreatitis and hypercalcemia after admission. She required renal support therapy (hemofiltration) after receiving antihypercalcemic therapy including vigorous rehydration, loop diuretics, calcitonin, and bisphosphonates. As for primary disease, the primary hyperparathyroidism was considered first based on the elevation of PTH and the nodule in the lower pole of the left thyroid lobe. Serum calcium (14.16mg/dl, reference range: 8.52-10.80mg/dl) and PTH (2586pg/ml, reference range: 12-69pg/ml) remained elevated after cervical exploration and the excision of the nodule, which showed typical thyroid follicle clusters and was diagnosed as solitary nodular goiter.

The vaginal bleeding of the patient was observed during stay. The physical examination identified enlargement of uterine as the size of a 16-week gestation. And thereafter the elevated calcium and PTH was suspected to be associated with gynecological tumors. A distinct elevation of CA125 was observed (287.9U/L-519.0U/L). Pelvic MRI imaging showed an enlarged uterus with disrupted junctional zone, thickened myometrium, and abnormal signals in the uterine cavity (**Figure 1C**). Adnexal masses and multiple enlarged lymph nodes were also observed. PET-CT showed increased SUVmax of 15.7 in an irregularly enlarged uterine (**Figure 1D**) and multiple lymph nodes along the uterus, abdominal aorta, and bilateral common iliac arteries (SUVmax 15.5). There was no radiographic evidence of bone metastasis.

**Figure 2:**
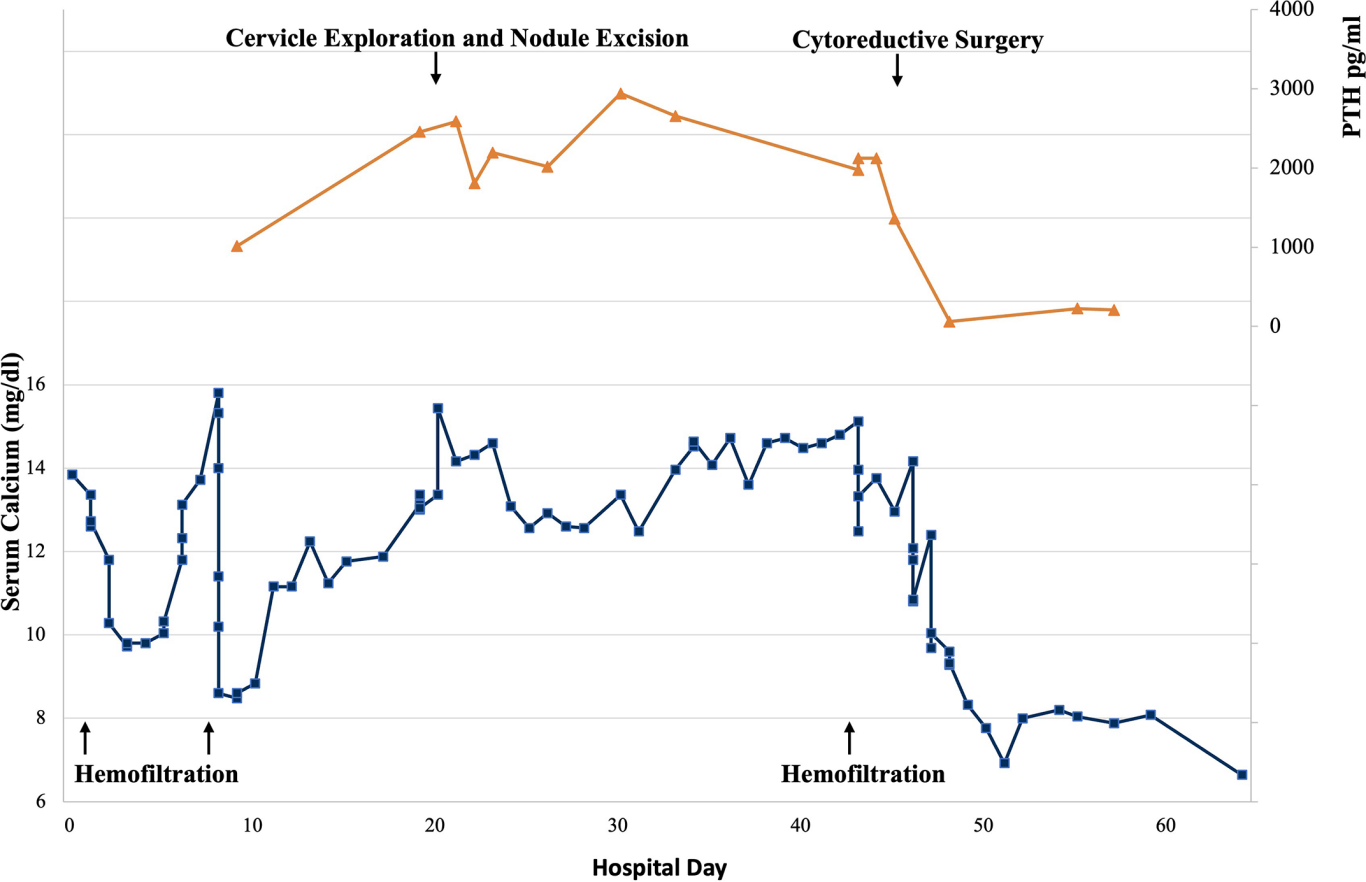
Serum calcium and parathyroid hormone (PTH) levels during patient’s hospitalization. Reference range of serum calcium: 8.52-10.80mg/dl, reference range of PTH: 12-69pg/ml.

The pathology of diagnostic curettage showed poorly-differentiated carcinoma with PTH expression, which suggested the hypercalcemia may attribute to PTH secreting by malignancy. The patient underwent a cytoreductive surgery (CRS, including total hysterectomy en bloc, bilateral adnexectomy, bilateral ovarian artery ligation, bilateral internal iliac artery ligation, appendectomy, omentectomy, and pelvic and para-aortic lymph node dissection). The level of serum calcium and PTH dropped immediately to normal range after surgery (calcium: 9.6mg/dl, reference range: 8.52-10.80mg/dl; PTH: 60pg/ml, reference range: 12-69pg/ml) (**Figure 2**).

**Figure 3:**
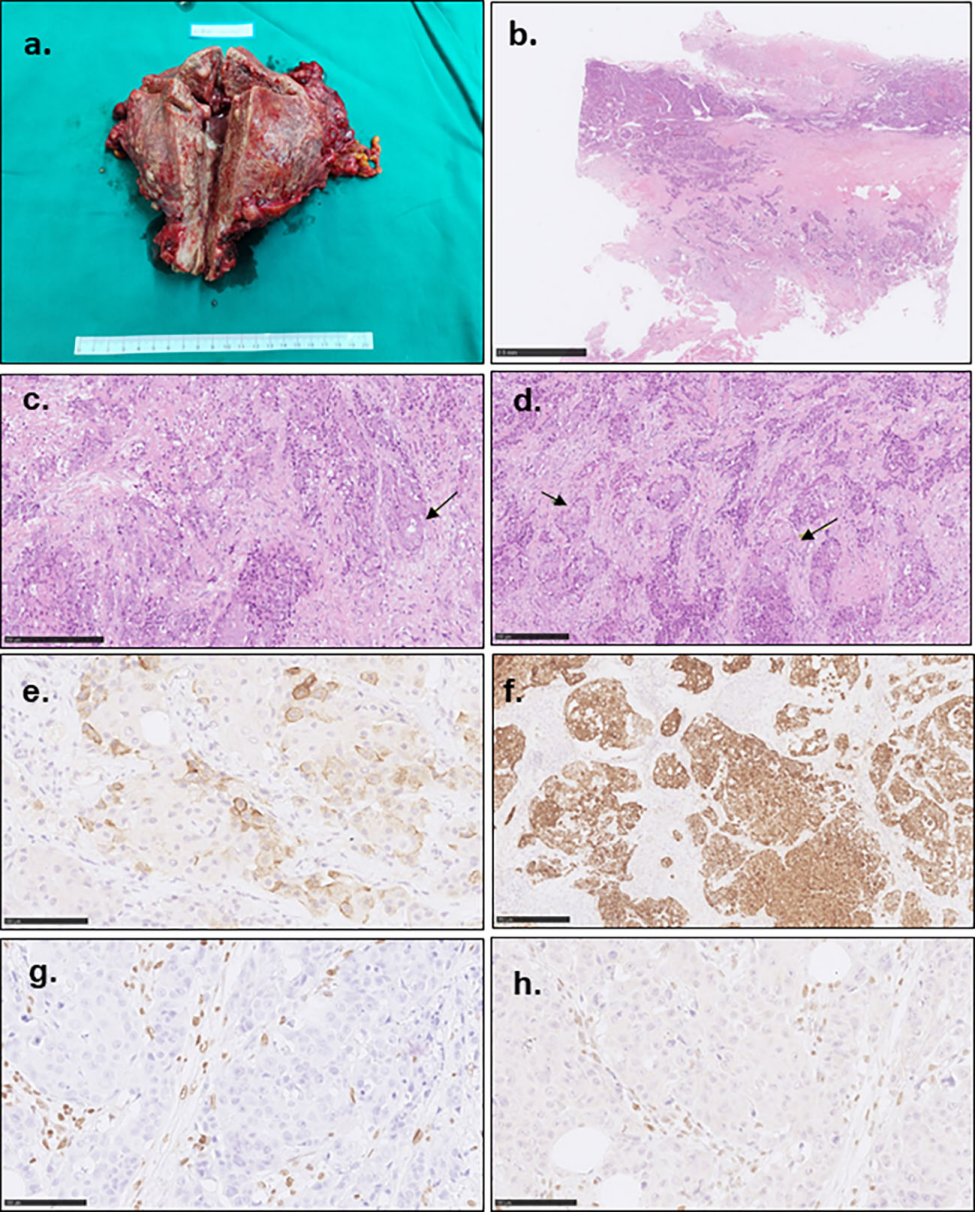
Gross photograph and pathological characteristics of the tumor. **(A)** showed the enlarged uterus. The endometrium was irregularly demolished, and the uterine wall and serous membrane were invaded. **(B)** showed the poorly differentiated carcinoma cells invaded the whole uterine wall with multiple embolisms. **(C)** indicated the poorly differentiated carcinoma with focal glandular formation (arrow). **(D)** showed focal squamous differentiation (arrow). **(E)** showed focal PTH staining. **(F)** showed diffusely AE1/AE3 staining. The tumor cells showed MLH-1 **(G)** and PMS-2 **(H)** negative staining contrast to the mesenchymal cells.

The excised specimen grossly showed the uterus enlarged to 15.1×13.2×7.3cm (**Figure 3A**). The endometrium was diffusely demolished, and the myometrium was uniformly thickened with necrosis and hemorrhage. The tumor invaded the perimetrium and cervix, and involved bilateral fallopian tubes, ovaries, rectum, sigmoid colon, the peritoneal reflection of bladder, and omentum. Histologically, the carcinoma was high grade, and focally showed both glandular and squamous differentiation (**Figures 3B–D**). In accordance with the curettage specimen, the carcinoma cells stained positive for PTH (**Figure 2E**), as while diffusely positive for AE1/AE3 (**Figure 3F**), MSH2 and MSH-6, focally positive for CK7, CK5/6 and CK34βe, and negative for Vimentin, S-100, LCA, MPO, SALL-4, Calcitonin, MLH-1 (**Figure 3G**) and PMS-2 (**Figure 3H**). The family history was negative for Lynch syndrome, and NGS found neither MLH-1 nor PMS-2 mutation. The final diagnosis was high grade MMR-deficient endometrioid carcinoma (**Figure 3**).

After CRS, this patient received four-course chemotherapy (3-course TP, paclitaxel and cisplatin; 1-course TC, paclitaxel and carboplatin) and three-course immunotherapy (Sintilimab). She died 5 months later after CRS due to multiple organ failure caused by tumor progression with serum PTH elevation.

## 3 Discussion

The patient who had PTH-secreting endometrial carcinoma is rare in MAH patients, and the differential diagnosis was challenging for clinicians because of concomitant nodular goiter mimic parathyroid tumors. In all cases of hypercalcemia in cancer, coexisting primary hyperparathyroidism should be routinely ruled out. The diagnosis of concomitant primary hyperparathyroidism was supported by elevated PTH and 99m Tc-MIBG positive nodule in the lower pole of the left thyroid lobe at first, which was later excluded because the pathology showed nodular goiter and no improvement after removal of the nodule. Therefore, the diagnosis of MAH was considered next.

Four pathogenic mechanisms have been demonstrated in MAH (1). Humoral hypercalcemia of malignancy is the most common cause of MAH resulted from tumor secreting PTHrP, which enhanced bone resorption and renal retention of calcium (1, 5, 6). Osteoclastic bone resorption increase is also a common cause, typically seen in bone metastasis (e.g., breast cancer) and multiple myeloma (7, 8). Specific lymphoma secreting active form of vitamin D (1,25-(OH)_2_D) is a less common cause of MAH (9). Ectopic secretion of authentic PTH by tumor is a rare cause, with only 35 cases has been previously documented, including one case of endometrial carcinoma (**Supplementary Table 1**) (4, 10–25). A low-normal or suppressed PTH level could be overserved in most cases due to the physiologic response to hypercalcemia, except for patients with ectopic secretion of PTH. In this case, an elevated PTH level, PTH-stained tumor tissue, and the reduction of calcium and PTH after CTRS supported the postulation that endometrial carcinoma secreting PTH is the most likely cause of hypercalcemia. Serum PTH was measured with two antibodies simultaneously in our case targeting N terminus (1-37) and C terminus (38-84). It is unlikely that PTHrP was measured, which has sequentially homologous to PTH in the N-terminal region from amino acids 1 to 13.

The detection of hypercalcemia in patients with malignancy usually denotes poor prognosis, and antihypercalcemic therapy has an important palliative role in alleviating symptoms (26, 27). Our patients received antihypercalcemic therapy including vigorous rehydration, aggressive calciuresis with loop diuretics, and inhibition of bone resorption with bisphosphonates and calcitonin. However, the serum calcium level remained high (11.24-15.80mg/dl, reference range: 8.52-10.80mg/dl) and required renal support therapy to control calcium level and prepare for surgery. In this case, the calcium and PTH level dropped immediately to normal range after CRS, and the patient received four-course chemotherapy (3-course TP and 1-course TC) and three-course immunotherapy (Sintilimab). Unfortunately, the patient died 5 months later.

Hypercalcemia caused by malignancy-secreting PTH is rare and prone to misdiagnosis. Management includes antihypercalcemic therapy and treatment of malignancy. Although rare, recognizing the association between elevated ectopic PTH-associated hypercalcemia and malignancy may prompt clinical investigation into detecting a potential underlying cancer in patients excluded from concomitant primary hyperparathyroidism.

## Data availability statement

The original contributions presented in the study are included in the article/**Supplementary Material**. Further inquiries can be directed to the corresponding authors.

## Ethics statement

The studies involving human participants were reviewed and approved by Institutional Ethics Committee of Peking Union Medical College Hospital. The patients/participants provided their written informed consent to participate in this study.

## Author contributions

All authors contributed significantly to this work. HL: Data acquisition, Visualization, Writing – original draft, Funding acquisition. DG: Data acquisition, Writing – original draft. YHua: Writing – original draft. JL: Data acquisition, Visualization. MZ: Data acquisition. ZL: Data acquisition. YHu: Data acquisition. TW: Data acquisition. YZ: Conceptualization, Data acquisition, Supervision, Writing – review & editing. WW: Conceptualization, Data acquisition, Funding acquisition, Supervision, Writing – review & editing. DC: Data acquisition, Supervision, Writing – review & editing. All authors approved this manuscript prior to submission and approved the submitted version.
