# Supplementary material for: Case Report: Life-threatening hypercalcemia associated with MMR-deficient endometrial carcinoma secreting parathyroid hormone
Source: Front Endocrinol (Lausanne). 2023 Jan 31;14:1125822. doi: 10.3389/fendo.2023.1125822 (PMC9927209; doi:10.3389/fendo.2023.1125822)
Supplement: Supplementary file 1 [file Table_1.docx]

Supplementary Material

Case Report: Life-threatening Hypercalcemia Associated with MMR-deficient Endometrial Carcinoma Secreting Parathyroid Hormone

Huazhen Liu^1,2, †^, Dan Gao^3, †^, Yongfa Huang^4, 5^, Ji Li^1,6^, Mengyun Zhao^7^, Zhaohui Lu^1,6^, Ya Hu^8^, Tao Wang^9^, Wenze Wang^1,6,*^, Yingqiang Zhang^10,11,*^, Dongyan Cao^9,*^

*** Correspondence:** Wang: wwzvssxy@126.com; Yingqiang Zhang: lemontreegarden@sohu.com; Dongyan Cao: caodongyan@pumch.cn.

# Supplementary Table 1 Malignancies with ectopic parathyroid hormone

| **Year** | **Author** | **Age/**  **Gender** | **Malignancy** | | | **Serum** | | | **Treatment for Malignancy** | **Prognosis (survival since hypercalcemia)** |
| --- | --- | --- | --- | --- | --- | --- | --- | --- | --- | --- |
|  |  |  | **Site** | **Pathology** | **Metastasis** | **Ca** | **P** | **PTH** |  |  |
| 1970 | Buckle *et al.*^1^ | 58y/F | Kidney | Adenocarcinoma, comprising clear and eosinophilic cells with pleomorphism and many mitoses | No | 13mg/dl | 2.9mg/dl | 0.6ng/ml (RR: <0.1-0.25ng/ml) | Surgery | NA |
| 1971 | Mavligit *et al.*^2^ | 44y/F | Rightsupra-acetabular | Metastatic breast carcinoma | Yes | 16mg/dl | 4mg/dl | 18.1ng/ml in tumor extract | Surgery, radiotherapy, androgen therapy, chemotherapy | 1 month |
| 1976 | Deftos *et al.*^3^ | 27y/F | Stomach | Carcinoid tumor | Yes | Elevated | NA | 4000pg/ml (RR: <800pg/ml) | Chemotherapy | 18 months |
|  |  | 37y/F | Pancreas | Islet cell carcinoma | Yes | 9.3mg/dl | NA | ~900pg/ml | Chemotherapy | NA |
| 1976 | Grajower *et al.*^4^ | 80y/M | Esophagus | Carcinoma | NA | 10.8mg/dl | 2.3mg/dl | 48uEq/ml | NA | Death during hospitalization |
| 1976 | Robin *et al.*^5^ | 66y/M | Intestine | Metastatic leiomyosarcoma | Yes | 11.4mg/dl | 3.6mg/dl | 255pg/ml | NA | 21 days |
| 1976 | Zidar *et al.*^6^ | 25y/M | Blood | Acute myeloblastic leukemia | NA | 12.3mg/dl | 2.2mg/dl | 301pg/ml (RR: <220pg/ml) | Chemotherapy | 25 months |
| 1980 | Hoeg *et al.*^7^ | 50y/F | Cervix | Invasive epidermoid carcinoma | NA | 14.6mg/dl | 2.6mg/dl | 60μLEq/ml (RR: 2-10μLEq/ml) | Radium implantation, external irradiation. | 2 months |
| 1983 | Samaan *et al.*^8^ | 49y/M | Tonsil | Squamous carcinoma | Yes | 10mg/dl | 3.4mg/dl | 0.95ng/ml (RR: 0.05-1.0ng/ml) | Surgery, chemotherapy | More than 5 years |
| 1984 | Mayes *et al.*^9^ | 6m/M | Kidney | malignant rhabdoid tumor (Rhabdoid Wilm's tumor) | Yes | 21.6mg/dl | 3.2mg/dl | 92 ulEq/ml (normal: 10ulEq/ml) | Surgery, chemotherapy | 12 days |
| 1989 | Yoshimoto *et al.* ^10^ | 70y/M | Lung | Small cell carcinoma | Yes | 17.48 mg/dl | NA | 9900ng/L (RR: <1300ng/L) | NA | 2 days |
| 1990 | Nussbaum *et al.*^11^ | 74y/F | Ovary | Adenocarcinoma | No | 15.6mg/dl | 2.1mg/dl | 325ng/L (RR: 10-60ng/L) | Surgery, chemotherapy | Last follow-up: 4 months (alive) |
| 1991 | Buller *et al.*^12^ | 72y/F | Uterus | Endometrial adenosquamous carcinoma | Yes | 14.9mg/dl | 3.9mg/dl | 345pg/ml (RR: 50-340pg/ml) | Surgery | NA |
| 1993 | Strewler *et al.*^13^ | 60y/M | Bone | Primitive neuroectodermal malignancy | Yes | 14.88mg/dl | NA | 90-290ng/L (RR: 15-65ng/L) | Biopsy | Last follow-up: 5 months |
| 1994 | Rizzoli *et al.*^14^ | 25y/M | Thymus | Thymoma | No | 11.6mg/dl | 1.9mg/dl | 8.9pmol/L (RR: 1.0-6.0pmol/L) | Surgery | NA |
| 1996 | Nielsen *et al.*^15^ | 71y/M | Lung | Squamous cell carcinoma | Yes | 10mg/dl | 1.1mg/dl | 150-560ng/L (RR: 10-50ng/L) | Surgery | 3 weeks |
| 1998 | Iguchi *et al.*^16^ | 72y/M | Thyroid | Papillary adenocarcinoma | No | 11.5mg/dl | 2.0mg/dl | 9800pg/ml (RR: 160-520pg/ml) | Surgery | 48 months (not relevant to thyroid adenocarcinoma) |
| 1999 | Koyama *et al.*^17^ | 83y/M | Liver | Hepatocellular carcinoma | No | 13.0mg/dl | 1.9mg/dl | 360mg/ml (RR: 15-50pg/ml) | Chemoembolization | Last follow-up: 24 months (alive) |
| 2002 | Uchimura *et al.*^18^ | 74y/M | Lung | Squamous cell carcinoma | Yes | 17.6mg/dl | NA | 180pg/ml (RR: 15-50pg/ml) | NA | Died and received autopsy |
| 2004 | Eid *et al.*^19^ | 73y/M | Bladder | Transitional cell carcinoma | Yes | 11.1mg/dl | NA | 397pg/ml | Chemotherapy | 39 months |
| 2004 | Ohira *et al.*^20^ | 33y/F | Ovary | Non-small cell neuroendocrine carcinoma admixed with endometrioid adenocarcinoma | Yes | 17.3mg/dl | NA | 205pg/ml (RR: 14-66pg/ml) | Surgery, chemotherapy | 6 months |
| 2005 | Chen *et al.*^21^ | 37y/F | Ovary | Small cell carcinoma | No | 8.6mg/dl | NA | ~95pg/ml (RR: 10-65.9pg/ml) | Surgery, chemotherapy | Last follow-up: 27 months (alive) |
| 2005 | Vacher-Coponat *et al.*^22^ | 59y/M | Pancreas | Neuroendocrine tumor | Yes | 22.4mg/dl | 4.7mg/dl | 394pg/ml (8-50pg/ml) | Chemotherapy | Last follow-up: 6 months (alive) |
| 2005 | Wong *et al.*^23^ | 62y/M | Nasopharynx | Rhabdomyosarcoma | Yes | 15.6mg/dl | 2.7mg/dl | 62.22pmol/L (RR: 0.839-1.453mmol/L) | Chemotherapy, radiotherapy | 2 months |
| 2006 | VanHouten *et al.*^24^ | 74y/F | Pancreas | Neuroendocrine tumor | Yes | 18mg/dl | NA | 2310pg/ml (RR: 6-40pg/ml) | NA | Several days |
| 2006 | Mahoney *et al.*^25^ | 72y/M | Liver | Hepatocellular carcinoma | No | 14.5mg/dl | 2.3mg/dl | 92pg/ml (RR: 12-65pg/ml) | Surgery, chemotherapy | Died |
| 2006 | Weiss *et al.*^26^ | 71y/F | Lung | Adenocarcinoma | No | 18.8mg/dl | 1.2mg/dl | 288pg/ml (RR: 10-65pg/ml) | Surgery | Last follow-up: 24 months (alive) |
| 2009 | Demura *et al.*^27^ | 43y/F | Thyroid | Medullary cancer | Yes | 9.2mg/dl | 3.5mg/dl | 82.7pg/ml (12-60pg/ml) | Surgery | NA |
| 2011 | Abe *et al.*^28^ | 73y/F | Liver | Hepatocellular carcinoma with a possible endocrine component | Yes | 12.9mg/dl | NA | 99pg/ml | Chemoembolization | 2 months |
| 2011 | Kandil *et al.*^29^ | 73y/F | Thyrothymic ligament | Neuroendocrine tumor (No parathyroid tissue), diagnosis: parathyroid lipoadenoma? | No | 10.6mg/dl |  | 126pg/ml | Surgery | Last follow-up: 6 months (alive) |
| 2013 | Nakajima *et al.*^30^ | 70y/M | Stomach | Adenocarcinoma | Yes | 14.8mg/dl | 1.8mg/dl | 190pg/ml (RR: 9-39pg/ml) | Chemotherapy | 1.5 months |
| 2013 | Doyle *et al.*^31^ | 28y/F | Pancreas | Neuroendocrine carcinoma (small-cell type) | Yes | 16.4mg/dl | 4.0mg/dl | 24.6pmol/L (RR: 1.6-9.3pmol/L) | Chemotherapy | 15 months |
| 2018 | Brooks *et al.*^32^ | 14y/M | Mandible | Metastatic alveolar rhabdomyosarcoma | Yes | 15.6mg/dl | 4.0mg/dl | 10.2pmol/L | Chemotherapy, | Last follow-up: 18 months |
| 2019 | Deshaies *et al.*^33^ | 53y/F | Pelvic | Recurrent endometrioid carcinoma with carcinosarcoma | Yes | 15.2mg/dl | NA | 1100pg/dl | Surgery | Several months |
| 2020 | Chen *et al.*^34^ | 76y/M | Paratracheal area | Metastatic prostate adenocarcinoma | Yes | Elevated | NA | 589.1pg/ml (RR: below 72pg/ml) | Surgery, androgen deprivation therapy | NA |

PTH: parathyroid hormone; RR: reference range; F: female; M: male;

1. Buckle RM, McMillan M, Mallinson C. Ectopic secretion of parathyroid hormone by a renal adenocarcinoma in a patient with hypercalcaemia. *Br Med J* 1970;4(5737):724-6. doi: 10.1136/bmj.4.5737.724 [published Online First: 1970/12/19]

2. Mavligit GM, Cohen JL, Sherwood LM. Ectopic production of parathyroid hormone by carcinoma of the breast. *N Engl J Med* 1971;285(3):154-6. doi: 10.1056/nejm197107152850307 [published Online First: 1971/07/15]

3. Deftos LJ, McMillan PJ, Sartiano GP, et al. Simultaneous ectopic production of parathyroid hormone and calcitonin. *Metabolism* 1976;25(5):543-50. doi: 10.1016/0026-0495(76)90008-1 [published Online First: 1976/05/01]

4. Grajower M, Barzel US. Ectopic hyperparathyroidism (pseudohyperparathyroidism) in esophageal malignancy. Report of a case and a review of the literature. *Am J Med* 1976;61(1):134-5. doi: 10.1016/0002-9343(76)90055-3 [published Online First: 1976/07/01]

5. Robin NI, Siegel LM, Hawker CD, et al. Hypercalcemia and metastatic intestinal leiomyosarcoma: a case of ectopic parathyroid hormone production. *Conn Med* 1976;40(9):609-11. [published Online First: 1976/09/01]

6. Zidar BL, Shadduck RK, Winkelstein A, et al. Acute myelobalstic leukemia and hypercalcemia. A case of probable ectopic parathyroid hormone production. *N Engl J Med* 1976;295(13):692-4. doi: 10.1056/nejm197609232951302 [published Online First: 1976/09/23]

7. Hoeg JM, Slatopolsky E. Cervical carcinoma and ectopic hyperparathyroidism. *Arch Intern Med* 1980;140(4):569-71. [published Online First: 1980/04/01]

8. Samaan NA, Ordonez NG, Ibanez ML, et al. Ectopic parathyroid hormone production by a squamous carcinoma of the tonsil. *Arch Otolaryngol* 1983;109(2):91-4. doi: 10.1001/archotol.1983.00800160025006 [published Online First: 1983/02/01]

9. Mayes LC, Kasselberg AG, Roloff JS, et al. Hypercalcemia associated with immunoreactive parathyroid hormone in a malignant rhabdoid tumor of the kidney (rhabdoid Wilms' tumor). *Cancer* 1984;54(5):882-4. doi: 10.1002/1097-0142(19840901)54:5<882::aid-cncr2820540521>3.0.co;2-n [published Online First: 1984/09/01]

10. Yoshimoto K, Yamasaki R, Sakai H, et al. Ectopic production of parathyroid hormone by small cell lung cancer in a patient with hypercalcemia. *J Clin Endocrinol Metab* 1989;68(5):976-81. doi: 10.1210/jcem-68-5-976 [published Online First: 1989/05/01]

11. Nussbaum SR, Gaz RD, Arnold A. Hypercalcemia and ectopic secretion of parathyroid hormone by an ovarian carcinoma with rearrangement of the gene for parathyroid hormone. *N Engl J Med* 1990;323(19):1324-8. doi: 10.1056/nejm199011083231907 [published Online First: 1990/11/08]

12. Buller R, Taylor K, Burg AC, et al. Paraneoplastic hypercalcemia associated with adenosquamous carcinoma of the endometrium. *Gynecol Oncol* 1991;40(1):95-8. doi: 10.1016/0090-8258(91)90095-m [published Online First: 1991/01/01]

13. Strewler GJ, Budayr AA, Clark OH, et al. Production of parathyroid hormone by a malignant nonparathyroid tumor in a hypercalcemic patient. *J Clin Endocrinol Metab* 1993;76(5):1373-5. doi: 10.1210/jcem.76.5.7684395 [published Online First: 1993/05/01]

14. Rizzoli R, Pache JC, Didierjean L, et al. A thymoma as a cause of true ectopic hyperparathyroidism. *J Clin Endocrinol Metab* 1994;79(3):912-5. doi: 10.1210/jcem.79.3.8077382 [published Online First: 1994/09/01]

15. Nielsen PK, Rasmussen AK, Feldt-Rasmussen U, et al. Ectopic production of intact parathyroid hormone by a squamous cell lung carcinoma in vivo and in vitro. *J Clin Endocrinol Metab* 1996;81(10):3793-6. doi: 10.1210/jcem.81.10.8855839 [published Online First: 1996/10/01]

16. Iguchi H, Miyagi C, Tomita K, et al. Hypercalcemia caused by ectopic production of parathyroid hormone in a patient with papillary adenocarcinoma of the thyroid gland. *J Clin Endocrinol Metab* 1998;83(8):2653-7. doi: 10.1210/jcem.83.8.5025 [published Online First: 1998/08/26]

17. Koyama Y, Ishijima H, Ishibashi A, et al. Intact PTH-producing hepatocellular carcinoma treated by transcatheter arterial embolization. *Abdom Imaging* 1999;24(2):144-6. doi: 10.1007/s002619900463 [published Online First: 1999/02/19]

18. Uchimura K, Mokuno T, Nagasaka A, et al. Lung cancer associated with hypercalcemia induced by concurrently elevated parathyroid hormone and parathyroid hormone-related protein levels. *Metabolism* 2002;51(7):871-5. doi: 10.1053/meta.2002.33341 [published Online First: 2002/06/22]

19. Eid W, Wheeler TM, Sharma MD. Recurrent hypercalcemia due to ectopic production of parathyroid hormone-related protein and intact parathyroid hormone in a single patient with multiple malignancies. *Endocr Pract* 2004;10(2):125-8. doi: 10.4158/ep.10.2.125 [published Online First: 2004/07/17]

20. Ohira S, Itoh K, Shiozawa T, et al. Ovarian non-small cell neuroendocrine carcinoma with paraneoplastic parathyroid hormone-related hypercalcemia. *Int J Gynecol Pathol* 2004;23(4):393-7. doi: 10.1097/01.pgp.0000139655.18062.12 [published Online First: 2004/09/24]

21. Chen L, Dinh TA, Haque A. Small cell carcinoma of the ovary with hypercalcemia and ectopic parathyroid hormone production. *Arch Pathol Lab Med* 2005;129(4):531-3. doi: 10.1043/1543-2165(2005)129<531:Sccoto>2.0.Co;2 [published Online First: 2005/03/30]

22. Vacher-Coponat H, Opris A, Denizot A, et al. Hypercalcaemia induced by excessive parathyroid hormone secretion in a patient with a neuroendocrine tumour. *Nephrol Dial Transplant* 2005;20(12):2832-5. doi: 10.1093/ndt/gfi065 [published Online First: 2005/09/29]

23. Wong K, Tsuda S, Mukai R, et al. Parathyroid hormone expression in a patient with metastatic nasopharyngeal rhabdomyosarcoma and hypercalcemia. *Endocrine* 2005;27(1):83-6. doi: 10.1385/endo:27:1:083 [published Online First: 2005/08/04]

24. VanHouten JN, Yu N, Rimm D, et al. Hypercalcemia of malignancy due to ectopic transactivation of the parathyroid hormone gene. *J Clin Endocrinol Metab* 2006;91(2):580-3. doi: 10.1210/jc.2005-2095 [published Online First: 2005/11/03]

25. Mahoney EJ, Monchik JM, Donatini G, et al. Life-threatening hypercalcemia from a hepatocellular carcinoma secreting intact parathyroid hormone: localization by sestamibi single-photon emission computed tomographic imaging. *Endocr Pract* 2006;12(3):302-6. doi: 10.4158/ep.12.3.302 [published Online First: 2006/06/15]

26. Weiss ES, Doty J, Brock MV, et al. A case of ectopic parathyroid hormone production by a pulmonary neoplasm. *J Thorac Cardiovasc Surg* 2006;131(4):923-4. doi: 10.1016/j.jtcvs.2005.12.020 [published Online First: 2006/04/04]

27. Demura M, Yoneda T, Wang F, et al. Ectopic production of parathyroid hormone in a patient with sporadic medullary thyroid cancer. *Endocr J* 2010;57(2):161-70. doi: 10.1507/endocrj.k09e-131 [published Online First: 2009/12/03]

28. Abe Y, Makiyama H, Fujita Y, et al. Severe hypercalcemia associated with hepatocellular carcinoma secreting intact parathyroid hormone: a case report. *Intern Med* 2011;50(4):329-33. doi: 10.2169/internalmedicine.50.4389 [published Online First: 2011/02/18]

29. Kandil E, Noureldine S, Khalek MA, et al. Ectopic secretion of parathyroid hormone in a neuroendocrine tumor: a case report and review of the literature. *Int J Clin Exp Med* 2011;4(3):234-40. [published Online First: 2011/10/07]

30. Nakajima K, Tamai M, Okaniwa S, et al. Humoral hypercalcemia associated with gastric carcinoma secreting parathyroid hormone: a case report and review of the literature. *Endocr J* 2013;60(5):557-62. doi: 10.1507/endocrj.ej12-0406 [published Online First: 2013/01/11]

31. Doyle MA, Malcolm JC. An unusual case of malignancy-related hypercalcemia. *Int J Gen Med* 2013;7:21-7. doi: 10.2147/ijgm.S51302 [published Online First: 2013/12/20]

32. Brooks R, Lord C, Davies JH, et al. Hypercalcaemia secondary to ectopic parathyroid hormone expression in an adolescent with metastatic alveolar rhabdomyosarcoma. *Pediatr Blood Cancer* 2018;65(1) doi: 10.1002/pbc.26778 [published Online First: 2017/08/27]

33. Deshaies D, Hariri N, Dyer B, et al. Life-Threatening Hypercalcemia due to Ectopic Intact Parathyroid Hormone Secretion from a Poorly Differentiated Endometrioid Carcinoma. *Am Surg* 2019;85(1):e45-e46. [published Online First: 2019/02/15]

34. Chen YH, Hsu YH, Chen HT, et al. Metastatic Parathyroid Hormone-Secreting Prostate Adenocarcinoma Mimicking Ectopic Parathyroid Adenoma Demonstrated on 99mTc-MIBI Image. *Clin Nucl Med* 2020;45(7):555-56. doi: 10.1097/rlu.0000000000003064 [published Online First: 2020/05/27]
